# How Do the Different Humor Styles of Streamers Affect Consumer Repurchase Intentions?

**DOI:** 10.3390/bs15040544

**Published:** 2025-04-17

**Authors:** Guangming Li, Yuan Xia

**Affiliations:** Business School, Hohai University, Nanjing 210000, China; 20120029@hhu.edu.cn

**Keywords:** e-commerce live streaming, humor style, relationship quality, social exchange theory, trust

## Abstract

With the rapid development of e-commerce live streaming, streamers play a crucial role in consumers’ shopping experience and decision-making. In this context, humor has gradually attracted widespread attention in the field of marketing as a communication strategy to enhance interaction between streamers and consumers. According to social exchange theory, this study specifically explores the differing impacts of e-commerce streamers’ humor styles on relationship quality, as well as the positive effect of relationship quality on consumers’ repurchase intention. Data were collected via an online survey with 519 valid responses, and structural equation modeling (SEM) was conducted using AMOS. The results reveal that affiliative humor significantly enhances consumers’ trust (β = 0.22, *p* < 0.001), which positively affects satisfaction and commitment, ultimately increasing repurchase intention. In contrast, aggressive humor undermines trust (β = −0.63, *p* < 0.001), leading to lower repurchase intention. This study provides theoretical support for e-commerce streamers to enhance consumers’ repurchase intentions by increasing the use of affiliative humor and reducing the use of aggressive humor.

## 1. Introduction

With the rapid development of e-commerce live streaming, the styles of streamers are becoming increasingly diverse. For example, Chinese streamers like Luo Yonghao attract viewers by telling funny stories or jokes. In contrast, streamers such as Xiao Yangge catch viewers’ attention by sarcasm and mutual teasing at each other. According to TikTok, the two streamers ranked 13th and 15th on the live sales ranking for July 2024, which is not a significant difference. This phenomenon raises critical questions regarding the effects of different humor styles in the context of e-commerce live streaming.

In contemporary academia, humor has attracted growing scholarly attention as a way to enhance relationship quality (Bergeron & Vachon, 2008), team cohesion (Martin et al., 2003), creativity (Lussier et al., 2017), and leadership (Romero & Cruthirds, 2006). Scholars in a variety of fields agree that humor has positive effects on personal health and relationships, including psychology (Martin et al., 2003), medicine (Martin, 2002), organizational behavior(Amos et al., 2014), and human resource management (Robert et al., 2016). Even though humor has demonstrated strong potential in fostering relationships, the application of humor in the marketing environment has been mainly focused on advertising (Du et al., 2023; Y. Huang, 2020), while relatively little research has been conducted in relationship marketing.

Social exchange theory suggests that individuals engage in different types of resource exchanges and that such exchanges can lead to the development of high-quality relationships. Cropanzano and Mitchell (2005) categorize resources into economic and socio-emotional resources. As a form of socio-emotional resource (Cooper et al., 2018), humor can help foster such relationships during the exchange process (Lambe et al., 2001). In the field of marketing, Wagle (1985) first suggested that humor may enhance buyer–seller relationships in commercial transactions and called for further research. Therefore, Lussier et al. (2017) proposed that a salesperson’s humor can foster lasting customer trust based on broadening and building theory. These studies provide a theoretical basis for the positive role of humor in enhancing customer trust and sales performance.

Despite the growing number of scholars conducting research in the field of marketing, a critical issue remains unresolved: humor is a multidimensional concept (Martin et al., 2003) that is often narrowly represented by positive humor (constructive humor), thereby failing to capture the differential effects of various humor styles. For example, Lussier et al. (2017) focused on the impact of constructive humor, which is typically positive and friendly. Thus, its impact on interpersonal relationships is usually positive. However, humor is a multifaceted construct that encompasses both constructive and potentially detrimental dimensions. Negative humor styles are aggressive or harmful, potentially resulting in adverse effects on personal relationships (Martin et al., 2003). Overall, this study will focus on e-commerce live streaming contexts and delve into the effects of different humor styles (positive and negative) on relationship quality.

In the highly competitive environment of e-commerce live streaming, streamers must not only encourage consumers to make initial purchases but also focus on fostering repeat purchases, which are essential for ensuring long-term sustainability (Zhang et al., 2011). Building long-term and stable relationships with consumers has become a key pathway to increasing customer loyalty and driving repurchase behavior (Morgan & Hunt, 1994). Streamers are thus encouraged to actively engage with their audiences to strengthen relationship quality and foster consumer loyalty (N. H. Jin et al., 2011).

Overall, this study employed a questionnaire-based survey to randomly sample live-streaming users in China. The aim was to examine the impact of different humor styles on relationship quality, as well as the positive effect of relationship quality on consumers’ repurchase intention.

## 2. Literature Review

Humor is a widespread interpersonal phenomenon that plays an important role in social interactions (Treger et al., 2013). While humor is commonly regarded as a positive phenomenon in previous research, some scholars have argued that it can be a double-edged construct (Lyttle, 2007; Malone, 1980). To offer a more comprehensive explanation, the humor styles theory proposed by Martin et al. (2003) categorizes humor along two dimensions: whether it is positive or negative, and whether it is directed toward others or oneself. Based on these distinctions, humor is divided into affiliative humor, self-enhancing humor, aggressive humor, and self-defeating humor. Affiliative humor is a non-hostile and tolerant form of humor in which the individual pleases others by telling funny stories or jokes, thereby alleviating and reducing interpersonal tension (Flaherty & Lefcourt, 2002). Self-enhancing humor refers to an outlook on life’s inconsistencies and contradictions humorously, even in the face of stress or adversity (Martin et al., 2003). Both styles of humor are associated with concepts of mental health and well-being, such as self-esteem or positive emotions (Samson & Gross, 2012).

Aggressive humor encompasses sarcasm, teasing, ridicule, derision, “put-down”, or disparagement humor (Zillmann, 1983). It aims to embarrass and ridicule individuals by denigrating them (Vinson, 2006). This humor style relates to the tendency to express humor without regard to its potential impact on others (Martin et al., 1993). Self-defeating humor is a type of humor that seeks to please others by excessively degrading oneself (Martin et al., 1993). Although people with self-defeating humor may be considered witty or funny, there is an element of emotional neediness, avoidance, and low self-esteem behind their use of humor (Fabrizi & Pollio, 1987). These two negative styles of humor have been shown to decrease relationship satisfaction and mental health (Samson & Gross, 2012).

The self-pointing humor styles focus primarily on individual displays of humor that enhance or undermine the self and may reduce perceived empathy from the consumer’s perspective. The others-pointing humor styles, on the other hand, focus more on individuals enhancing or damaging their relationships with others.

Due to the virtual and interactive nature of e-commerce live streaming (M. Huang et al., 2023; Y. Liu et al., 2020), streamers need to interact frequently and closely with consumers to enhance engagement. In this interactive context, the use of self-pointing humor styles (self-enhancing humor and self-defeating humor) is more relatively constrained in comparison to others-pointing humor styles (affiliative humor and aggressive humor). Consumers’ perceptions of a streamer’s humorous behavior are primarily formed through the interactions between the streamer and their audience, specifically through the humor exhibited in interpersonal interactions (Shi et al., 2017). Therefore, this study focuses on the theoretical and practical value of others-pointing humor in e-commerce live streaming. Specifically, in e-commerce live streaming, affiliative humor is characterized by the live streamer sharing funny remarks or jokes, and aggressive humor is manifested by the live streamer making sarcastic, threatening, or demeaning remarks to the guests or viewers during the live stream (Martin et al., 2003).

Relationship quality proposed by Crosby, Evans, and Cowles in 1990 is the degree to which the customer relies on the honesty and trust of the salesperson’s future behavior based on past satisfaction (Crosby et al., 1990). Building on this foundation, various scholars have redefined relationship quality based on the characteristics of different industry contexts. For example, relationship quality is defined as the perceived evaluation of the service perceived by the customer in the relationship compared to some intrinsic or extrinsic quality standards in the service industry (Liljander & Strandvik, 1995). Johnson (1999) defines relationship quality among marketing channel members as the overall depth and atmosphere of the members’ relationship. Combining the research of many scholars on relationship quality, Chinese scholars R. H. Liu and Yao (2005) defined relationship quality as the common cognitive evaluation of the degree to which the relationship fulfills the mutual needs of both parties according to certain criteria. In essence, it refers to a set of intangible benefits that can increase the value of what the enterprise provides, strengthen the trust and commitment of both parties in the relationship, and maintain a long-lasting relationship (R. H. Liu & Yao, 2005).

### 2.1. Humor Style and Relationship Quality

Based on the characteristics of socio-emotional resources, and the fact that humor conveys a sense of support and friendship (Blau, 1964; Cropanzano & Mitchell, 2005), humor is an important socio-emotional resource (Cooper et al., 2018). According to social exchange theory, humor can build quality relationships during resource exchange (Lambe et al., 2001). When streamers entertain the audience with lighthearted or affiliative humor, it indicates an intention to establish a positive exchange with trading partners (Pundt & Herrmann, 2015). Using humor wisely can be a tool for developing and building lasting relationships (Cooper, 2005).

However, all of the above research focuses on positive humor, which is a style of humor that is widely believed to lead to positive outcomes (Lussier et al., 2017; Treger et al., 2013). As most scholars pointed out, humor has not only positive but also negative aspects (Lyttle, 2007; Malone, 1980). Humor is a multifaceted structure (Martin et al., 2003). Therefore, this study posits that distinct humor styles produce divergent effects on relationship quality in transactional settings. Negative humor, which can be demeaning, hurtful, or sarcastic towards oneself or others, may offend the transactional counterpart and diminish the overall quality of the relationship (Martin et al., 2003).

There is considerable debate in the academic community regarding the dimensions of relationship quality. In the context of retailing, Crosby et al. (1990) argue that relationship quality is a high-level construct that includes at least two dimensions of trust and satisfaction. Smith (1998) suggests that relationship quality in purchasing and supply should include at least three dimensions: satisfaction, trust, and commitment. Hennig-Thurau and Klee (1997) found that relationship quality should encompass perceived overall quality, trust, and commitment. Undoubtedly, trust, satisfaction, and commitment are fundamental dimensions of relationship quality regardless of industry context (R. H. Liu & Yao, 2005). This study adopts the widely accepted view that trust, satisfaction, and commitment constitute the three dimensions of relationship quality.

Trust is defined as the perceived confidence in the reliability and honesty of trading partners (Morgan & Hunt, 1994). In e-commerce live streaming, consumers encounter various uncertainties (Crosby et al., 1990) due to its social, immediate, and virtual characteristics (M. Huang et al., 2023); thus, trust plays an important role. In the field of organizational behavior, Karakowsky et al. (2019) revealed the mechanism of affiliative humor in the construction of leadership trust through empirical research. Specifically, they found that affiliative humor influences trust in leaders by conveying both self-confidence and kindness to organizational members. Similarly, previous research suggests that positive humor from salespeople can increase customer trust (Lussier et al., 2017). Unfortunately, they did not explore the relationship between negative humor and trust. Aggressive humor is when leaders try to satirize others to boost their ego, and it is clear that aggressive humor signals a lack of benevolence (Neves & Karagonlar, 2020). Trustworthiness and benevolence are fundamental dimensions of trust (Ganesan, 1994). Thus, this study explores the relationship between different humor styles of streamers and consumer trust in the e-commerce live streaming domain.

### 2.2. Trust, Satisfaction, and Commitment

Trust, satisfaction, and commitment are the core dimensions of relationship quality. Prior studies have found that there is a causal linkage between these three dimensions (P. L. Huang et al., 2008). Therefore, this study tries to sort out the relationship between the three dimensions. Satisfaction refers to a state of psychological contentment that arises from an overall evaluation of the transactional experience (P. L. Huang et al., 2008). In management, trust is a good predictor of satisfaction (Driscoll, 1978). Trust reduces uncertainty (Mayer et al., 1995) and mitigates opportunistic behavior among members (Wicks et al., 1999). The current literature has explored the relationship between trust and satisfaction (Ganesan, 1994; Selnes, 1998). Driscoll (1978) proposed and confirmed that the more trust one has in an organization, the more likely one is to be satisfied with the organization-based trust theory. In the field of marketing, the positive and significant effect of trust on satisfaction has also been confirmed (Lee et al., 2008). Therefore, this study hypothesizes that in e-commerce live streaming, the more consumers trust the streamers, the more likely they are to be satisfied with the streamers.

Commitment is defined as a partner’s belief among relationship members that it is important to maintain an ongoing relationship with the other member and thus will do their best to maintain the relationship (Morgan & Hunt, 1994). Commitment reveals the tendency of both parties in a relationship to stay or leave (P. L. Huang et al., 2008). Therefore, commitment is a key variable in measuring relationship quality (Dwyer et al., 1987).

Trust enhances expectations of mutual loyalty between trading partners (P. L. Huang et al., 2008). Social exchange theory views the relationship between trust and commitment as mutually beneficial for both trading partners (Cropanzano & Mitchell, 2005). Mistrust reduces relational commitment and leads to the switching and leaving of trading partners (McDonald, 1981). According to Commitment–Trust Theory proposed by (Morgan & Hunt, 1994), trust affects commitment positively. Ganesan (1994) suggests that high levels of trust among trading partners lead to a greater focus on long-term relationship development. Numerous empirical studies have provided empirical evidence supporting the role of trust in fostering commitment. For instance, Chumpitaz and Paparoidamis (2007) confirmed that trust exerts a positive influence on commitment. Similarly, the positive effect of trust on commitment has also been validated within the context of e-commerce (Li et al., 2020). It has also been pointed out that commitment often implies a certain amount of dedication and sacrifice. Commitment then only arises when trust is generated. Thus, trust is regarded as an antecedent variable of commitment (Garbarino & Johnson, 1999).

### 2.3. Relationship Quality and Repurchase Intention

In the field of relationship marketing, scholars generally agree that customers’ post-purchase behaviors, such as repurchase and customer retention, are the result of high-quality relationships (Roberts et al., 2003). Previous studies have confirmed the positive impact of relationship quality on repurchase intention. Similarly, Zhang et al. (2011) found a positive correlation between relationship quality and repurchase intention in the context of e-commerce.

Repurchase intention is widely recognized as a consequence of customer satisfaction (Oliver, 1980). Previous research has confirmed that the higher the satisfaction, the higher the repurchase intention of consumers (Davidow, 2003; Lee & Lin, 2005). Pappas et al. (2014) provided evidence of an association between satisfaction and repurchase intention through the use of the unified theory of acceptance and use of technology (UTAUT). In the context of online shopping, Hsu et al. (2006) suggested that customer satisfaction has a positive influence on repurchase intention. Similarly, in the context of e-commerce live streaming, the positive effect of satisfaction on repurchase intention has also been confirmed (Yang & Wang, 2021). Commitment is closely linked to loyalty (Bettencourt, 1997), and Oliver (1999) argues that repurchase intention is the attitudinal loyalty stage of customer loyalty. Many scholars have confirmed that commitment positively affects customer loyalty (Dwyer et al., 1987; Morgan & Hunt, 1994). Chou and Chen (2018) confirmed in their study that commitment has a significant positive effect on repurchase intention. Therefore, this study supposes that consumers’ commitment to streamers positively affects consumers’ repurchase intention in e-commerce live streaming.

### 2.4. The Current Study

This study examines the relationship between e-commerce streamers’ humor styles and consumers’ repurchase intention. Specifically, we predict the following:

**H1.**  *(a) Affiliative humor style is positively related to trust, while (b) aggressive humor style is negatively related to trust*.

**H2.**  *Trust is positively related to (a) satisfaction and (b) commitment*.

**H3.**  *Both (a) satisfaction and (b) commitment are positively related to repurchase intention*.

**Figure 1:**
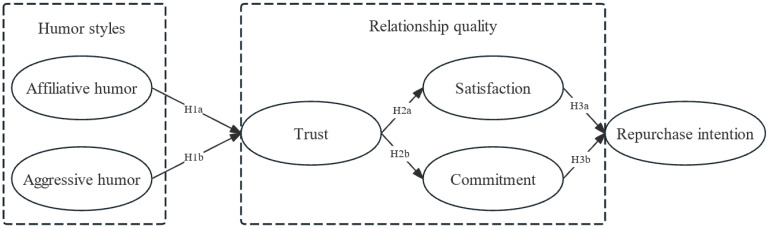
Proposed conceptual framework.

An illustrative representation of the proposed structural model is demonstrated in Figure 1.

## 3. Materials and Methods

### 3.1. Participants

The questionnaires were distributed randomly using the Credamo sample payment service, which is an industry-recognized questionnaire platform. IP control and the adoption rate of the subjects’ previous responses were used to make the selection of the subjects real and valid. Participants from various cities in China were asked to complete the questionnaire based on their most recent experience of watching live streaming. They received a cash prize of CNY 2 after completing the questionnaire. We received 638 questionnaires. After listwise deletion of the participants who had not watched live streaming and those who did not meet the screening questions, our final sample retained 519 (81.3%) valid questionnaires.

After statistically analyzing the data from 519 valid questionnaires, the demographics of the participants are shown in Table 1. There are 370 (71.3%) female and 149 (28.7%) male participants. The ratio can be explained by the fact that females are more engaged in live shopping. Most respondents (80.9%) were aged between 18 and 35. Over half of the respondents had a monthly income of CNY 6000 (60.5%). Nearly half of the respondents watched live streaming for 30–60 min (49.9%). Their educational attainment was less than high school 0.4%, high school 2.7%, junior college 6.2%, undergraduate degree 72.1%, and master or other postgraduate diploma 18.7%. The sample is basically consistent with the characteristics of online live streaming user groups and the duration of watching live streaming in the “2021 China Online Live Streaming Industry Development Research Report”, which is representative.

### 3.2. Measurement

All the scales were measured through Likert-type scales anchored by 1 = totally disagree and 7 = totally agree.

Streamer humor style: Using the humor style questionnaire (Martin et al., 2003), we measured two types of humor (affiliative and aggressive). We adapted the scale to focus on the streamers. Sample items included “The streamer often jokes with guests or viewers of his/her live stream” (affiliative humor) and “If the streamer dislikes a person, he/she often belittles that person with ridicule or derision” (aggressive humor). Cronbach’s alphas were 0.876 (affiliative humor) and 0.757 (aggressive humor).

Relationship quality: We measured 3 dimensions of trust, satisfaction, and commitment, drawing primarily on the research of Morgan and Hunt (1994) and Selnes (1993). Sample items included “The streamer is trustworthy” (trust), “Overall, the streamer satisfies me very much” (satisfaction), and “I will follow the streamer’s live events for a long time” (commitment). Cronbach’s alphas were 0.883 (trust), 0.857 (satisfaction), and 0.896 (commitment).

Consumers’ repurchase intentions: We refer to the research of Chiu et al. (2012) and Zeithaml et al. (1996). The sample items are “I will continue to purchase goods from the live stream in the future”, “I will give priority to the live stream for purchasing goods if I need them in the future”, and “I am willing to recommend the live stream to other friends and family”. Cronbach’s alpha was 0.860.

Control variables: We examined the respondents’ age, gender, education level, monthly income, and the length of time they watched a live stream at one time.

We invited several scholars to conduct multiple rounds of translation and revision of the questionnaire, and the revised version was also validated.

### 3.3. Common Method Variance and Confirmatory Factor Analysis

Given that all items in the same questionnaire were completed by the same respondent, the questionnaire in this study was set up to measure with anonymity, and the order of the questions was rationalized. In addition, this study uses Harman’s single-factor test to examine common method biases. The result showed that the first factor accounted for 23.494% (less than 40%) of all explanatory variables, which indicated that common method biases were not serious.

In this study, reliability analysis was conducted through SPSS 26.0. Specifically, the reliability of the questionnaire was examined by calculating Cronbach’s alpha. The reliability of each variable in this study reached an acceptable Cronbach’s alpha value of more than 0.7 (0.757–0.896) (Nunnally, 1967). This indicates that the internal consistency of the scales performed well. In addition, before testing our hypotheses, this study used 24.0 to test the scale for convergent and discriminant validity with confirmatory factor analysis (CFA). As shown in Table 2, the standardized factor loading coefficients for all question items were greater than 0.6 (0.669–0.895), which meets the item reliability test (Jolliffe, 1988). The results showed that the average variance extracted (AVE) for each latent variable was greater than 0.5 (0.521–0.745), and the composite reliability (CR) was greater than 0.7 (0.765–0.898), indicating that the scales of this study have good convergent validity (Fornell & Larcker, 1981). Finally, the discriminant validity test was conducted. The fact that the MaxR(H) (Maximum H Reliability) values are higher than the CR values means that discriminant validity is established (Fornell & Larcker, 1981). The diagonal line in Table 3 is the AVE square root value, and the remaining values are the correlation coefficients of the variables with each other. As can be seen from the table, the AVE square root value of each factor is greater than the absolute value of the correlation coefficient between that factor and the other factors, which indicates good discriminant validity (Fornell & Larcker, 1981).

## 4. Results

This study employs structural equation modeling (SEM) using AMOS 24.0 for hypothesis testing, as the aim is theory confirmation rather than prediction (Kline, 2016). A commonly used method in SEM is maximum likelihood estimation, which requires a sample size at least ten times the number of observed variables and data that conform a normal distribution (Hair et al., 2010). The valid sample size is 519, with 19 observed variables, and the ratio of the two is more than 10. The maximum absolute skewness of all observed variables is 1.288 (less than 3), and the maximum absolute kurtosis is 1.846 (less than 10), indicating that the sample data basically conform to the normal distribution, which can be analyzed in the next step (X. P. Jin & Bi, 2017). The chi-square value (χ^2^) of 336.761 (df = 145, *p* < 0.001) was obtained, which indicates the goodness of fit. In addition, the χ^2^/df ratio was 2.322, which is less than the recommended threshold of 3 (Marsh & Hocevar, 1988), which increases the acceptability of the model.

The goodness of fit index (GFI = 0.935) met the criterion of 0.9, showing that the model has an overall good fit and explains the observed data well. Similarly, the comparative fit index (CFI = 0.973), normed fit index (NFI = 0.954), and root mean square error of approximation (RMSEA = 0.051) were at an acceptable level, thus enhancing the reliability of the model.

**Figure 2:**
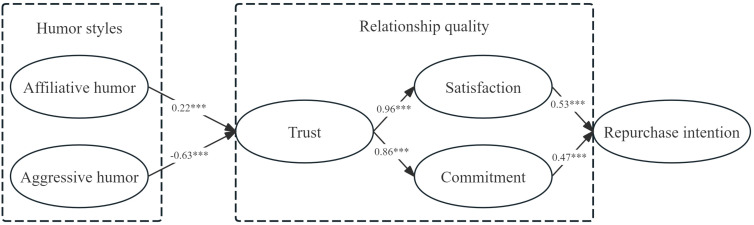
Structural modeling results. *** represent 1% significance level.

From the results of the structural model shown in Figure 2, we find that the path coefficient from affiliative humor to trust is 0.22 (*p* < 0.001), indicating a significant positive correlation between the streamer’s affiliative humor and consumer trust. Conversely, the path coefficient from aggressive humor to trust is −0.63 (*p* < 0.001), which indicates that the anchor’s mocking humor weakens the consumer’s trust, and there is a significant negative correlation. Therefore, hypotheses H1a and H1b are verified. The path coefficients from trust to satisfaction and commitment are 0.96 (*p* < 0.001) and 0.86 (*p* < 0.001), indicating that the more consumers trust the streamers, the higher their satisfaction and commitment will be. Consequently, both hypotheses H2a and H2b are also validated. Additionally, the path coefficients of satisfaction and commitment to repurchase intention are 0.53 (*p* < 0.001) and 0.47 (*p* < 0.001), respectively, indicating that the more satisfied consumers are with the anchors, the higher their repurchase intention is; similarly, commitment to the anchors positively affects repurchase intention. Therefore, hypotheses H3a and H3b are validated. Specific path test results and hypothesis tests are shown in Table 4.

## 5. Discussion

### 5.1. Theoretical Implications

This study provides a new perspective on the importance of the use of humor by streamers in e-commerce live streaming. Consistent with previous research, we find that affiliative humor of the streamers has a significant positive effect on consumer trust (H1a) (Lussier et al., 2017). Consumers tend to trust the streamers more when they use affiliative humor because this humor helps to improve the quality of the relationship between the streamers and consumers. This result echoes previous findings in the literature (Bompar et al., 2023), thus providing further evidence of the role of affinity humor in easing the relationship between streamers and consumers.

However, this study further emphasizes the importance of viewing humor as a multidimensional construct. In this sense, this study echoes the research of Bompar et al. (2023), who found that positive humor has a positive effect on relationship quality, but negative humor may deteriorate relationship quality. Unlike previous research that focused mostly on the positive humor dimension (Lussier et al., 2017), this study reveals that different types of humor produce very different effects in the marketing context and that aggressive humor in particular may have a negative effect on the relationship between streamers and consumers.

Thus, this study provides a more nuanced theoretical perspective on the role of humor and supports the idea of humor as a double-edged sword (Lyttle, 2007; Malone, 1980), as different types of humor can lead to significantly different or even opposite effects. By demonstrating the importance of different humor styles in building quality relationships between streamers and consumers, this research expands the effective selling literature and provides new theoretical support for the use of humor as a marketing tool. By analyzing the subtle effects of humor in the anchor–consumer relationship, we provide important theoretical insights for the effective use of humor in e-commerce live streaming in the future.

### 5.2. Practical Implications

E-commerce live streaming has emerged as a new marketing model, which has received widespread attention from enterprises and consumers. Based on the results of this study, the following recommendations are proposed.

This study explores the impact of streamer humor styles on consumers’ repurchase intentions. The results show that streamers’ humor styles promote repurchase intention by affecting the relationship quality between consumers and streamers. From a strategic perspective, e-commerce companies should specifically select streamers who exhibit affiliative humor for product or service promotion. Affiliative humor effectively enhances the streamer’s credibility, which in turn increases consumer satisfaction and commitment, ultimately leading to stronger repurchase intentions. Therefore, when selecting streamers, e-commerce companies should prioritize the alignment of humor styles to achieve higher consumer loyalty and repeat purchase rates in a competitive market.

As the e-commerce live streaming market continues to develop and mature, streamer types are showing a tendency towards homogenization. In this fiercely competitive landscape, attracting consumer attention becomes a key strategy for streamers to stand out. Currently, some streamers, such as Xiao Yangge, utilize teasing and sarcasm to engage viewers. However, according to this study’s findings, aggressive humor does not effectively enhance consumer trust. While such humor may attract short-term purchases, its failure to establish long-term trust undermines consumer satisfaction and commitment, hindering future repurchases. Consequently, streamers should favor affiliative humor, which can strengthen consumer trust by bridging emotional distances and alleviating interpersonal tensions.

For streamers, it is essential not only to adopt appropriate humor styles to gain consumer trust but also to focus on enhancing consumer satisfaction and demonstrating commitment. Consumers’ repurchase intention is critical for increasing loyalty and long-term profitability. Streamers can effectively build and maintain strong relationships with their audience by improving consumer satisfaction and promoting repeat purchases. Moreover, showing attentiveness to consumers’ needs and commitment during interactions will further solidify trust, creating a positive purchasing cycle. This strategy not only enhances the personal brand value of the streamer but also provides robust support for the overall performance growth of e-commerce enterprises.

### 5.3. Limitations and Future Research

Like all studies, this study has some limitations and opportunities for future research. First, the sample consists of a significantly higher number of female participants than male participants, which may affect the representativeness of the study results. Future research could further explore potential gender differences in humor perception and trust. Second, this study primarily focuses on the Chinese market; therefore, its findings may have regional limitations, making it difficult to directly generalize to other cultural or market environments. Future research could adopt a cross-cultural approach to further explore the impact of humor styles on consumer behavior across different regions and cultural backgrounds, thereby enhancing the generalizability of the findings. Third, there may be conceptual overlap between commitment and repurchase intention in this study, which could lead to bias in the structural equation modeling (SEM) path coefficients. Future studies could delve deeper into the relationships between these concepts, refine their theoretical implications, and avoid potential measurement errors or model bias. Fourth, this study primarily uses external assessments to verify the impact of streamer humor styles on consumer repurchase intention, but such external evaluations may not fully capture the subtle effects of humor. Therefore, future research should consider using self-assessment methods to evaluate the streamer’s humor style and consumers’ perceptions of the streamer. This approach will allow for a more detailed understanding of the role of humor style in the e-commerce live streaming context. By combining consumers’ direct experiences with streamers’ self-perceptions, researchers can better elucidate the role of humor in fostering consumer relationships and driving repurchase intentions. Finally, the impact of streamer humor styles on consumers’ repurchase intentions is a complex process. Future research could explore more potential mediating and moderating variables, such as consumer emotions, involvement, and others, to gain a more comprehensive understanding of this influence mechanism.

## 6. Conclusions

In the context of fierce competition in the e-commerce live streaming field, e-commerce companies and streamers need to recognize the key role of streamers in driving consumer purchasing behavior and adopt corresponding strategies to enhance the influence of streamers. This study proposes a theoretical model of the influence of different humor styles of e-commerce streamers on consumers’ repurchase intention. This study finds that the affiliative humor of the streamers can promote consumers’ trust in the streamers, but the aggressive humor of the streamers can significantly diminish this trust. Consumers’ trust has a significant positive effect on satisfaction and commitment, which in turn has a significant positive effect on consumers’ willingness to repurchase. This finding provides a new theoretical perspective for relationship marketing in digital commerce, where humor, as a relational signal, can effectively pull in the relationship between streamers and consumers and promote repurchase intention. E-commerce companies and anchors can appropriately adjust their current streamers’ styles based on this finding so as to gain consumers’ trust in a competitive market and thus optimize business performance.

## Figures and Tables

**Table 1:** Demographic summary.

Variable		Frequency	Percentage
Gender	Male	149	28.7%
	Female	370	71.3%
Age	Under 18	1	0.2%
	18–25	179	34.5%
	26–35	241	46.4%
	36–45	72	13.9%
	Above 45	26	5.0%
Education level	Less than high school	2	0.4%
	High school	14	2.7%
	Junior college	32	6.2%
	Undergraduate degree	374	72.1%
	Master or other postgraduate diploma	97	18.7%
Monthly income	Less than CNY 2000	72	13.9%
	CNY 2001–4000	58	11.2%
	CNY 4001–6000	76	14.6%
	CNY 6001–8000	94	18.1%
	More than CNY 8001	219	42.4%
Length of time to watch live streaming	Less than 30 min	120	23.1%
	30–60 min	259	49.9%
	1–3 h	129	24.9%
	3–5 h	9	1.7%
	More than 5 h	2	0.4%

The sample did not include participants who had not watched live streaming or failed to meet the screening question.

**Table 2:** Standardized factor loading, AVE, CR, Cronbach’s α, and MaxR (H).

Factors		Standardized Factor Loading	AVE	Composite Reliability	Cronbach’s α	MaxR (H)
Affiliative humor	AF1	0.866	0.651	0.881	0.876	0.895
	AF2	0.746				
	AF3	0.734				
	AF4	0.870				
Aggressive humor	AG1	0.669	0.521	0.765	0.757	0.772
	AG2	0.775				
	AG3	0.718				
Trust	TR1	0.867	0.720	0.885	0.883	0.889
	TR2	0.806				
	TR3	0.844				
Satisfaction	SA1	0.776	0.666	0.856	0.857	0.860
	SA2	0.829				
	SA3	0.849				
Commitment	CO1	0.813	0.745	0.898	0.896	0.904
	CO2	0.881				
	CO3	0.895				
Repurchase intention	RE1	0.857	0.681	0.865	0.860	0.869
	RE2	0.787				
	RE3	0.829				

AF represents affiliative humor, AG represents aggressive humor, TR represents trust, SA represents satisfaction, CO represents commitment, and RE represents repurchase intention.

**Table 3:** Discriminant validity.

	Affiliative Humor	Aggressive Humor	Trust	Satisfaction	Commitment	Repurchase Intention
Affiliative humor	0.801					
Aggressive humor	−0.432 ***	0.72				
Trust	0.448 ***	−0.62 ***	0.847			
Satisfaction	0.471 ***	−0.596 ***	0.822 ***	0.817		
Commitment	0.500 ***	−0.512 ***	0.757 ***	0.757 ***	0.865	
Repurchase intention	0.464 ***	−0.59 ***	0.771 ***	0.806 ***	0.808 ***	0.826

*** represents 1% significance level, and the diagonal digit is the root value of the AVE for the factor.

**Table 4:** Specific path test results and hypotheses tests.

Hypotheses	Paths	Estimate	Standardized Estimate	Standard Error	Critical Ratio	*p*	Results
H1a	Affiliative humor → Trust	0.200	0.222	0.044	4.518	***	√
H1b	Aggressive humor → Trust	−0.675	−0.628	0.064	−10.545	***	√
H2a	Trust → Satisfaction	0.883	0.962	0.043	20.683	***	√
H2b	Trust → Commitment	1.178	0.862	0.054	21.805	***	√
H3a	Satisfaction → Repurchase intention	0.599	0.531	0.068	8.778	***	√
H3b	Commitment → Repurchase intention	0.356	0.470	0.044	8.112	***	√

*** represents 1% significance level.

## Data Availability

The original contributions presented in this study are included in the article. Further inquiries can be directed to the corresponding author.
